# Advancing Telesurgery Connectivity Between North and South America: the first Remote Surgery Conducted Between Orlando and São Paulo in Animal Models

**DOI:** 10.1590/S1677-5538.IBJU.2024.0601

**Published:** 2025-01-10

**Authors:** Marcio Covas Moschovas, Shady Saikali, Mischa Dohler, Ela Patel, Travis Rogers, Ahmed Gamal, Jeffrey Marquinez, Vipul Patel

**Affiliations:** 1 AdventHealth Global Robotics Institute FL United States AdventHealth Global Robotics Institute, FL, United States; 2 University of Central Florida FL United States University of Central Florida – UCF, FL, United States; 3 Ericsson Inc. Advanced Technology Group Santa Clara United States Advanced Technology Group, Ericsson Inc., Santa Clara, United States

## COMMENT

The 2001 Lindbergh operation, telesurgery between Strasbourg (France) and New York (USA), was a milestone in surgery history, showing the feasibility of transatlantic telesurgery and the beginning of the robotic surgery era. However, despite this success, the widespread adoption of remote surgery was limited for many years by technological limitations and economic constraints (1, 2). In this context, advancements in robotic systems, 5G networks, and fiber-optic infrastructure have reignited interest in this area (3–5). The rapid advancement of telesurgery technology presents an unprecedented opportunity to address global healthcare disparities and revolutionize surgical education (6). However, despite this technological progress, the literature still lacks articles and studies about long-distance remote surgeries using these new technologies (7).

In this scenario, since December 2023, our group has been performing trials and remote connections with several centers worldwide (8, 9). Consequently, we conducted a pioneering telesurgery procedure connecting São Paulo (Brazil) to Orlando (USA) on September 3rd, 2024. In this trial, the animal model (live porcine) was located in Orlando, while the surgeon (MCM) operated remotely from São Paulo using the MicroPort® MedBot™ Toumai robotic platform (Shanghai MicroPort MedBot Group Co., Ltd.). This milestone achievement marks the first telesurgery robotic procedure between these two cities located in opposite parts of America (North and South) and opens new avenues for future studies on remote surgical connectivity inside the American Continent. Moreover, it underscores the immense potential that telesurgery holds in transforming global healthcare delivery, approximating the expertise of remote surgeons to improve operative outcomes, as addressed by our team in previous articles (5).

Before conducting our study, the research project received approval from the Nicholson Center's Animal Care Committee (Celebration, FL, USA). All procedures strictly adhered to institutional ethical standards and regulatory requirements, following the Institutional Animal Care and Use Committee (IACUC) guidelines (10). The study followed the EQUATOR and ARRIVE reporting guidelines for research involving animal models (11). One surgeon from our team (MCM) traveled to São Paulo, Brazil to operate on the console, while our team stayed in Orlando (USA) to manage the patient cart and porcine model. Under general anesthesia, the animal was initially positioned in right lateral decubitus, and the robotic trocars were placed along the paramedian abdominal line, as routinely done in robotic surgery training in animal models. We used four robotic trocars, and one assistant trocar positioned between the second and third robotic trocars. Instruments, including scissors, bipolar forceps, and Cadiere forceps, were inserted under direct visualization.

The procedure began with dissection of the left renal hilum, isolating the renal artery and vein, which were then clamped with Bulldog clamps for warm ischemia. A round defect was created on the anterior surface of the kidney, followed by suturing of the parenchyma using barbed sutures, secured with hem-o-lok clips at the edges. Once the suture was complete, the Bulldog clamps were removed, hemostasis was confirmed, and a radical nephrectomy was performed by ligating the renal artery and vein with hem-o-lok clips. The same steps were performed on the contralateral kidney. Total operative time was 35 minutes on the right side (warm ischemia 12 minutes) and 37 on the left (warm ischemia 13 minutes). No complications or robotic faults were recorded during the procedure. The verbal communication between both centers was also optimal, and surgeons could control the robot independently from each center's console during several surgery steps, highlighting the tremendous potential of telesurgery in minimizing complications and training robotic surgeons remotely.

**Figure 1:**
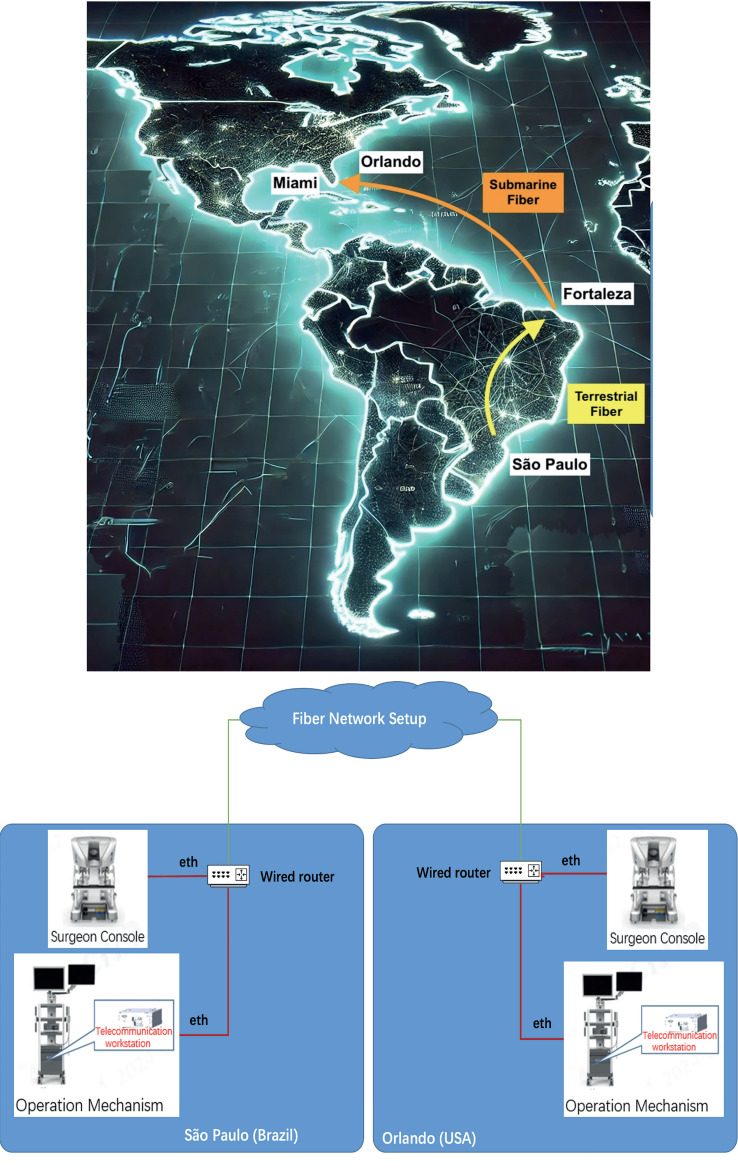
Trail-SP-Orlando.

It is essential to emphasize that distance plays a critical role in assessing the optimal telesurgery connection between two centers. In our study, the fiber distance between São Paulo and Orlando is approximately 8,000 kilometers, resulting in a round-trip signal distance of nearly 16,000 kilometers. Given that the speed of light through fiber-optic cables is around 200,000 km/s, the theoretical minimum round-trip time (RTT) due to distance alone is approximately 80 milliseconds. Our study used corporate Ethernet in the hospitals, then terrestrial fiber between hospitals and landing stations (i.e. Orlando – Miami and Fortaleza – Sao Paulo); and the middle-segment was covered by submarine fiber (Miami – Fortaleza), illustrated in Figure-1. The average round-trip of 120 ms (118-150 ms) with no frame loss, indicating high connection quality. Note that telesurgery latency is influenced by more than just distance, as other network variables also play a role. No intraoperative complications or robotic malfunctions occurred during the procedures, demonstrating the system's reliability and the optimal performance of the Toumai robot under these conditions.

Considering that Brazil is a continental country, the fifth biggest area in the world, with several remote and underserved areas, the humanitarian implications of telesurgery are profound. Currently, millions of individuals in underserved regions of the world lack access to timely, high-quality surgical care. Geographical barriers, a shortage of skilled healthcare professionals, and limited infrastructure contribute to significant disparities in healthcare access, eventually leading to suboptimal surgical outcomes in these areas. In this scenario, the new platforms with telesurgery capabilities have the potential to mitigate these barriers by enabling expert surgeons to perform complex procedures remotely, offering their skills and expertise to regions where local healthcare systems may be insufficient (7). This technology can effectively bridge the gap between patients and advanced medical care, bringing critical surgical interventions, small procedures, and even biopsies to those who need them most, regardless of their location.

In this context, several articles in the literature described the benefits of Telementoring and Teleproctoring in surgical training and emergency scenarios (12, 13). Remote surgical interventions could be lifesaving in situations where access to care is restricted by natural disasters, conflict zones, resource-limited settings, and areas such as neurovascular, cardiology, and oncology, where time to intervention is crucial. As technology evolves, telesurgery could become an essential tool for global humanitarian missions, helping to alleviate the burden of surgical diseases in remote and low-resource areas (2).

In addition to its humanitarian impact, telesurgery holds significant promise in medical education. Traditionally, surgical training has been limited by geographical proximity and the availability of experienced mentors (6). With the advent of remote surgery, these constraints are diminished, allowing for real-time collaboration and mentorship between surgeons in different parts of the world simultaneously. Now, expert surgeons can supervise, guide, and assist in complex procedures remotely, fostering a global community of surgical practices. This creates opportunities for knowledge exchange and skill enhancement that were previously unattainable. Medical institutions worldwide could leverage telesurgery for remote proctoring and training programs, enabling students and professionals to observe or participate in surgeries performed by leading experts without the need for physical relocation. This opportunity will also minimize the travel of experts, proctors, and invited professors to perform cases in other cities because these connections will be enabled by a robot with telesurgery capabilities. Furthermore, patients will save resources by having the opportunity to undergo a surgical procedure in their city next to their families instead of traveling to other centers or paying for the expenses of a proctor travelling from a different city or country.

Our telesurgery study between São Paulo and Orlando serves as a compelling example of how this technology can be applied in practice with optimal connectivity. During the procedure, the surgeon in São Paulo could remotely control the surgical robot in Orlando, maintaining optimal communication with the local team. This real-time collaboration between teams on different continents exemplifies the potential for international surgical partnerships in several surgical fields. More importantly, it highlights how remote surgery can overcome geographical limitations, offering the possibility of expert care and mentorship access across vast distances.

While the initial success of this procedure is promising, the implementation of telesurgery on a global scale will require further advancements in technology, robust telecommunications infrastructure, and transparent regulatory frameworks. In this context, a collaborative community of experts should be involved in the implementation, maintenance and responsible evolution of remote surgery following optimal ethical standards (7). This community involves medical societies, medical councils, engineers, surgeons, robotic companies, telecommunication entities, local governments, legal experts, and all stakeholders involved with remote procedures. Before any remote surgery, it is crucial to have these specialists and stakeholders involved in consenting and orienting the patient regarding the next steps of that surgical approach. The establishment of standardized protocols and guidelines for remote surgery will be critical to ensure patient safety and maintaining the quality of care (7, 14, 15). Furthermore, latency, data security, and interoperability remain key challenges that must be addressed by experts to ensure safe and effective implementation. In this context, after our extensive experience with telesurgery in Asia operating in several centers, our collaborative community created the 10 commandments for a safe and ethical exploration of telesurgery (Table-1) (7).

**Table 1:** Telesurgery collaborative community 10 commandments for the ethical and safe exploration of telesurgery.

1. **Prioritize Patient Safety and Efficacy:** Ensure that all robotic and networking systems meet the highest standards of safety and efficacy through rigorous testing, certification, and regular monitoring. Ensure that all medical staff have adequate training and certification in telesurgery

2. **Maintain Transparency, Honesty & Consent:** Provide clear, truthful, and accessible information about the capabilities, limitations, risks and economics associated with robotic telesurgery to patients and the public. Obtain, explicit consent about the remote nature of the surgery and the teams involved.

3. **Foster Human Interaction and Empathy**: Despite the remote delivery method of robotic telesurgery, ensure that human empathy and patient comfort are prioritized by involving compassionate medical staff in the patient care process.

4. **Adhere to Ethical Medical Practices**: Maintain the highest standards of medical ethics, including respecting patient autonomy, doing no harm, providing the best possible care and having telesurgicalapprovals from national, trans-national and international regulatory bodies. Initial telesurgeries ought to be performed under an IRB/investigational protocol monitored by an administrative body.

5. **Establish Clear Accountability and Liability**: Define and communicate the shared responsibility and liability among all parties involved in robotic telesurgery, including the local and remote surgical teams and hospitals, device manufacturers, and telecommunication providers.

6. **Uphold Data Privacy and Security**: Implement robust cybersecurity measures and adhere to international data protection laws to safeguard sensitive patient information stored, processed and transmitted in real-time against unauthorized access and breaches.

7. **Promote Accessibility and Equity**: Make robotic telesurgery accessible and affordable to all individuals, regardless of geographic location or socioeconomic status, to prevent healthcare disparities nationally and globally.

8. **Encourage International Collaboration and Standardization**: Work towards harmonizing regulatory and ethical telesurgerystandards across borders to facilitate international cooperation and consistency in robotic telesurgery practices.

9. **Promote Continuous Education and Training**: Ensure ongoing professional development and training for all medical professionals involved in robotic telesurgery to keep pace with technological advancements and ethical considerations.

10. **Promote Innovation of Safe & Trusted Telesurgery Technologies:** Ensure continuous innovation in telesurgery, robotics and connectivity technologies. Encouraging the development and application of emerging technologies such as artificial intelligence and real time surgical imaging.

Telesurgery, as previously mentioned, represents a transformative innovation with the potential to improve healthcare access, reduce disparities, and enhance surgical education worldwide. Our recent procedure between São Paulo (Brazil) and Orlando (USA) marks a significant step forward in the development of telesurgical capabilities between North and South America, demonstrating that such connections are not only feasible but also offer immense potential for future applications in several surgical fields. However, despite the optimism, we should acknowledge that several steps are yet to come until these results can be translated to human trials, such as guidelines, regulations, connectivity studies, and the involvement of local medical societies and committees for a safe and ethical application of remote surgery. By continuing to explore and refine this technology, we have the opportunity to create a more equitable healthcare system where advanced surgical care and training are available to all, irrespective of geographical constraints.
